# Practices of community pharmacists towards COVID-19 treatment in Vietnam: a simulated client study

**DOI:** 10.1186/s12913-026-15395-0

**Published:** 2026-08-18

**Authors:** Hoa Quoc Nguyen, Suong Thi-Thanh Duong, Phuong Bao Quan, Tuyen Thanh Du, Uyen Thao Uyen Truong, Nga TQ. Nguyen, Tuan-Dung Nguyen

**Affiliations:** 1 School of Pharmacy, University of Medicine and Pharmacy at Ho Chi Minh City, Ho Chi Minh City, Vietnam; 2 School of Pharmacy, Queen’s University Belfast, Belfast, UK

**Keywords:** Community pharmacists, COVID-19, Simulated client

## Abstract

**Background:**

Recent reports have raised concerns about drug overuse and misuse in self-medication practices for COVID-19 treatment. This study was aimed to assess the availability and appropriateness of medication dispensing provided by community pharmacists to COVID-19 patients in Ho Chi Minh City, Vietnam.

**Methods:**

This cross-sectional study used the simulated client method to examine 102 randomly selected community pharmacies from 13 central districts of Ho Chi Minh City between May 2022 and July 2022. Each pharmacy was visited by 2 simulated clients asking to buy medications for a relative with mild COVID-19 symptoms. Characteristics of pharmacies and pharmacists along with medications dispensed were recorded. Factors associated with appropriate medication transaction were explored using the Bayesian Model Averaging method.

**Results:**

Of 102 enrolled community pharmacies, 63 (61.8%) were independent. Most encountered pharmacists were female (90.2%) and were not pharmacists-in-charge (82%). Of 98 transactions with identified medications, cough medicines (93.9%), antipyretics (88.8%), and dietary supplements (80.6%) were frequently dispensed. Moreover, prescription-only medications dispensed without a medical prescription such as antibiotics (59.2%), corticosteroids (60.2%) and antivirals (7.1%), were also recorded. Additionally, the rate of antibiotics dispensed in chain pharmacies was significantly lower than that in independent pharmacies (OR = 0.29, 95% CI = 0.12–0.69).

**Conclusions:**

This study recorded high rates of antibiotics and corticosteroids for COVID-19 mild symptoms dispensed without a prescription. Further research is warranted to improve practices of community pharmacists towards COVID-19 treatment.

**Supplementary Information:**

The online version contains supplementary material available at https://doi.org/10.1186/s12913-026-15395-0.

## Background

Since the outbreak of the COVID-19 pandemic in 2020, healthcare systems around the world have been under tremendous pressure due to a shortage of medical supplies, a high mortality rate, and significant demands for medical care [1]. Therefore, the preparedness of a country’s healthcare system plays a crucial role in infection control and social stability during the COVID-19 pandemic. Some countries such as the United Kingdom, South Korea, or Thailand adapted promptly and efficiently to this crisis situation by enhancing their pharmaceutical services along with providing expanded guidelines and frameworks to ensure both public and healthcare worker safety [2–4].

In Vietnam, the healthcare system was overwhelmed with COVID-19 patients during the huge outbreak, especially in Ho Chi Minh City (HCMC) in 2021. To resolve this crisis, the government developed emergency guidance to help reduce the pressure on the frontline medical teams. Patients with mild to moderate COVID-19 were allowed to self-monitor at home and would be distributed medicine packages by local healthcare staff based on their symptoms [5, 6]. In addition, as pharmacies are the most accessible and the first point of contact in Vietnam [7], more than 6,500 pharmacies in HCMC were called upon to provide consultations and essentials for COVID-19 self-monitored patients at home [8]. The government and local authorities also kept updating information and guidelines for COVID-19 management not only for healthcare professionals but also for community [9].

While pharmacists in Vietnam may have received some training or guidance during the COVID-19 pandemic via online documents or webinars by the Ministry of Health or experienced healthcare professionals [9, 10], it is important to note that dispensing medications without a prescription, particularly antibiotics, was already a common practice before the pandemic [11]. There have been many reports on drug abuse and misuse in self-medication practices for COVID-19 prevention and treatment, resulting in unfortunate consequences [12]. This has raised concerns about the preparedness and responses of community pharmacists (CPs) to the COVID-19 pandemic; yet little work has been done to address this problem. This study was aimed to assess the availability and appropriateness of medication dispensing provided by CPs to COVID-19 patients using the simulated client (SC) method.

## Method

### Design and population

This cross-sectional study was conducted between May and July 2022 in thirteen central districts across HCMC where a total of 1683 pharmacies participated in the COVID-19 healthcare campaign [13]. This timeframe was selected because it followed the peak of the COVID-19 outbreak in HCMC in 2021, aligning with the study’s objective to assess pharmacist practices after the initial crisis period, when national and local COVID-19 treatment guidelines had been established.

It was assumed that the proportion of appropriate medication dispensing provided by CPs for COVID-19 self-monitoring patients with mild symptoms was 50% with a confidence interval of 95% and a standard error of 10%. The sample size for the study, which was determined by using the online software Raosoft^®^, was 91 community pharmacies.

A list of pharmacies, including both independent and chain pharmacies participating in the COVID-19 healthcare campaign, was obtained from the website of HCMC Department of Health [13]. Pharmacies from 13 central districts (Districts 1, 3, 4, 5, 6, 8, 10, 11, Binh Thanh, Go Vap, Phu Nhuan, Tan Binh, and Tan Phu) were randomly selected from the list. To account for attrition, the selected sample size for the study was 104 community pharmacies (8 pharmacies per district). These pharmacies were then coded, filtered by districts, and randomly selected using Microsoft Excel 2016 software to obtain the determined sample size. Pharmacies which were not open at the time of visit (temporarily closed or relocated) were excluded from the study.

### Data collection

The SC method, which involved training an individual to portray a specific patient/ client scenario during interaction with healthcare providers, was used to mitigate the Hawthorne effect; this approach ensured the subjects were not aware of being observed, thereby minimizing alterations in their behaviors [14]. Four pharmacy students (S.T-T.D, P.B.Q, T.T.D, U.U-T.T) were trained by the researchers (H.Q.N, T-D.N) to act as a SC with a standardized script to ensure the consistency of the representation. The preset scenario was established based on relevant guidance on COVID-19 treatment and prevention issued by the Vietnam Ministry of Health and other worldwide organizations [15–17]. The preset scenario included the following elements:

- The SCs would present themselves as family members concerned about a relative, a 32-year-old non-pregnant woman who had tested positive for COVID-19 via self-test the previous day.
- They would describe the relative’s symptoms – fever, dry cough, loss of smell and body aches lasting for 2 to 3 days – consistent with a mild presentation of COVID-19 and meeting the criteria for self-monitoring at home as per national guidelines.
- The SCs would seek the pharmacist’s advice on managing the symptoms without specifying any particular medications.

Each selected pharmacy was visited simultaneously by two students acting as family members of a COVID-19 patient with typical symptoms, seeking advice and medications from the pharmacist. One of the two students interacted with the CP, asking to buy medications for the simulated COVID-19 patient, while the other who acted as a companion discreetly took brief written notes on a smartphone (no audio or video recording or photography was performed). Afterwards, the two students documented each pharmacist’s responses using a standardized data collection form immediately after leaving the pharmacy to limit any possible recall bias.

The preset scenario and data collection form were developed by the research team and were evaluated by an expert in the field of clinical pharmacy and an experienced CP. The data collection form comprised four main sections: characteristics of pharmacy and CP, patient information collected by CP, dispensed medications, and other relevant information recorded. Furthermore, another CP with more than 10 years of experience also assisted the research team with the identification of unlabelled medications dispensed which could be encountered in some community pharmacies. Unlabelled medications refer to those dispensed without proper labelling, such as the drug name, dosage, or other essential information. This practice has been reported previously in community pharmacies in Vietnam [18–20].

The preset scenario and data collection form were also piloted with 10 pharmacies which were not included in the study. This helped with validating the scenario as well as assessing the appropriateness and feasibility of both the script and the data collection form.

### Data analysis

Data were analyzed using statistical packages of R software version 4.0.5. The study outcomes were appropriate medication transaction (yes/no), antibiotic dispensing (yes/no), and corticosteroid dispensing (yes/no). A medication transaction (or transaction) was defined as the complete set of medications that were either dispensed or recommended to the SCs during a single pharmacy visit. A transaction was deemed appropriate if all medications dispensed or recommended were appropriate, regardless of whether they were ultimately purchased or of the counselling provided. To determine the appropriateness of dispensed medications, we referred to the following national and local guidelines which were publicly available online via government websites and social media at the time of the study:

- Vietnam Ministry of Health’s guidelines on the management of COVID-19 patients at home (Decision No. 261/QD-BYT) [21],
- HCMC Department of Health’s guidelines on healthcare packages for COVID-19 patients at home [22].

Based on these guidelines, four groups of medications were considered appropriate to improve symptoms for the simulated COVID-19 patient, comprising cough medicines, antipyretics, dietary and electrolyte supplements; prescribed medications such as corticosteroids, antibiotics, antiviral drugs, and anticoagulants were deemed inappropriate. The percentage of appropriate medications dispensed in a transaction at each pharmacy was also recorded.

The characteristics of the study sample are presented as numbers (N) and percentages (%) for categorical variables, and medians and interquartile ranges (IQR) for continuous variables. The p-values were calculated with chi-square tests at an alpha level of 5%. We also explored the association between the three study outcomes (i.e. appropriate medication transaction, antibiotic dispensing, and corticosteroid dispensing) and the explanatory variables, comprising type of pharmacy, consultation length, CP’s gender, CP’s age, and CP’s identification [23, 24]. The Bayesian Model Averaging (BMA) analyses were conducted to account for model uncertainty and to identify the combination of predictors that best explained each study outcome [25, 26]. BMA estimates the posterior probability of each possible model and selects those with the highest likelihood of being the true model. Variables with a posterior inclusion probability greater than 50% were considered strongly associated with the outcome and were then included in the final multivariable logistic regression (p-values < 0.05 were considered significant) [27].

### Ethical considerations

This study was approved by the Research Ethical Committee at University of Medicine and Pharmacy at HCMC (No. 508/HDDD-DHYD). The research team obtained informed consent from all participating pharmacies one or two months prior to data collection. During this process, the study’s purpose, procedures, and the use of SCs were explained with assurances given regarding the confidentiality of any potentially identifiable data collected. The pharmacist-in-charge at each pharmacy, primarily responsible for professional activities, was identified using the list of pharmacies participating in the COVID-19 healthcare campaign, available on the HCMC Department of Health website [13]. During the visits, if a pharmacist other than the pharmacist-in-charge (identified via their name tag) dispensed the medications, the SCs proceeded with the interaction and recorded the data as usual. Information details about the selected pharmacies and the enrolled CPs was kept confidential in a password-protected laptop and was deleted after the study was completed.

## Results

Of 104 randomly selected pharmacies, 102 were visited by the SCs (the other two were found closed and excluded from the study). Most of the enrolled community pharmacies were independent (61.8%) and located on main roads (97.1%) (Table 1). Of 102 encountered pharmacists, 15 (14.7%) were identified as pharmacist-in-charge. The median consultation length was 8 (IQR: 5–9) minutes.

**Table 1:** Characteristics of visited pharmacies (*N* = 102)

Characteristics			*n* (%)
Encountered community pharmacist	Gender	female	92 (90.2)
		male	10 (9.8)
	Age	$\:\le\:$40 years old	77 (75.5)
		> 40 years old	25 (24.5)
	Identification	pharmacist-in-charge	15 (14.7)
		non-pharmacist-in-charge	82 (80.4)
		no identification	5 (4.9)
	Consultation length	< 5 min	34 (33.3)
		5–10 min	49 (48.0)
		> 10 min	19 (18.7)
Visited pharmacy	Type of pharmacy	independent	63 (61.8)
		chain	39 (38.2)
	Location	main road	99 (97.1)
		alley	3 (2.9)

### Medications dispensed by CPs

Of 102 pharmacies visited, one refused dispensing medications due to no confirmation letter (from a local authority or a doctor’s prescription for the simulated COVID-19 patient) provided when being asked. The median number of medications dispensed by CPs among the 101 pharmacies was 5 (IQR: 4–5). Moreover, medications dispensed with no labels were also recorded in 54/102 transactions (52.9%). The research team was able to identify unlabelled medications in 51 of these 54 transactions.

Among 98 transactions with all medications identified by the research team, the most frequently dispensed medications were cough medicines (93.9%), antipyretics (88.8%), and dietary supplements (80.6%), whereas antihistamines (27.6%), electrolyte supplements (7.1%), and antiviral drugs (7.1%) were less frequently dispensed (Table 2). None of the transactions comprised anticoagulants. Noticeably, antibiotics accounted for 59.2% of the 98 transactions, including penicillins (37.9%), cephalosporins (24.1%), macrolides (17.2%) and quinolones (3.5%). Amoxicillin/clavulanic acid was the most frequently dispensed antibiotic (20.6%). Moreover, corticosteroids accounted for 60.2% of the 98 transactions. Details of the medicines recommended by CPs are displayed in Table S1 (available in Additional file 1).

Thirteen (13.3%) of the 98 transactions with identified medications dispensed were deemed appropriate. The median percentage of appropriate medications dispensed at each pharmacy was 60% (IQR: 40.0%-72.9%).

**Table 2:** Summary of medications recommended by the pharmacists

Medications dispensed		*n* (%)
Antipyretics (*N* = 87)	Paracetamol	38 (43.7)
	Paracetamol-containing combinations	35 (40.2)
	Others*	14 (16.1)
Dietary supplements (*N* = 79)	Vitamin and minerals	60 (76.0)
	Others*	19 (24.1)
Cough medicines (*N* = 92)	Dry cough medicines	53 (57.6)
	Combination products	35 (38.0)
	Others*	4 (4.4)
Electrolyte supplements (*N* = 7)	Oral rehydration salts	7 (100)
Corticosteroids (*N* = 59)	Methylprednisolone	26 (44.1)
	Prednisolone	3 (5.1)
	Others*	30 (50.8)
Antibiotics (*N* = 58)	Penicillins	22 (37.9)
	Cephalosporins	14 (24.1)
	Macrolides	10 (17.3)
	Quinolones	2 (3.4)
	Others*	10 (17.3)
Antiviral drugs (*N* = 7)	Molnupiravir	7 (100)
Other medicines (*N* = 34)	Antihistamines	27 (79.4)
	Antacids	7 (20.6)

*Others: For detailed information on the medicines, please refer to Table S1 in Additional File 1

### Responses of CPs

Of the 102 pharmacies visited, relevant information collected by the CPs was recorded, including patient identification (53.9%), patient’s information and medical history (77.5%), and COVID-19-related information (70.6%). Moreover, 45.1% of the CPs inquired about other symptoms besides those described by the SCs. Regarding counseling information, 56 CPs (54.9%) provided general advice on COVID-19 to the SCs. However, only nine CPs (8.8%) provided information on emergency warning signs of COVID-19 during self-monitoring at home. Additionally, seven CPs (6.9%) counselled the SCs on undesirable effects of antivirals (molnupiravir) dispensed; none of the surveyed CPs provided information on usage or cautions of other dispensed medications.

### Factors associated with the dispensing practice of CPs

Characteristics of the explanatory variables by the three study outcomes are presented in Table 3.

**Table 3:** Characteristics of the explanatory variables by the three study outcomes

Explanatory variables^a^		Appropriate medication transaction			Transaction with corticosteroids dispensed			Transaction with antibiotic dispensed		
		Yes *N* (%)	No *N* (%)	*P* value^b^	Yes *N* (%)	No *N* (%)	*P* value^b^	Yes *N* (%)	No *N* (%)	*P* value^b^
Type of pharmacy (*N* = 98)	independent	5 (38.5)	54 (63.5)	0.157	39 (66.1)	20 (51.3)	0.209	41 (70.7)	18 (45.0)	**0.019**
	chain	8 (61.5)	31 (36.5)		20 (33.9)	19 (48.7)		17 (29.3)	22 (55.0)	
Gender of pharmacist (*N* = 98)	female	13 (100.0)	75 (88.2)	0.350	51 (86.4)	37 (94.9)	0.307	51 (87.9)	37 (92.5)	0.521
	male	0	10 (11.8)		8 (13.6)	2 (5.1)		7 (12.1)	3 (7.5)	
Age of pharmacist (*N* = 98)	$\:\le\:$40 years	10 (76.9)	65 (76.5)	1.000	46 (78.0)	29 (74.4)	0.866	44 (75.9)	31 (77.5)	1.000
	> 40 year	3 (23.1)	20 (23.5)		13 (22.0)	10 (25.6)		14 (24.1)	9 (22.5)	
Identification of pharmacist (*N* = 94)	pharmacist-in-charge	3 (23.1)	11 (13.6)	0.404	6 (10.7)	8 (21.1)	0.277	7 (12.5)	7 (18.4)	0.620
	non-pharmacist-in-charge	10 (76.9)	70 (86.4)		50 (89.3)	30 (78.9)		49 (87.5)	31 (81.6)	
Consultation length^c^ (*N* = 98)		8 (5–9)	8 (5–9)	0.631	8 (5–9)	9 (5–10)	0.635	8.5 (5–10)	9 (5–10)	0.118

^a^Chi square test was employed to test the association between two independent variables, except consultation length which was examined using Wilcoxon test

^b^P value < 0.05 was considered significant

There were 94 transactions with all the explanatory variables fully recorded. These transactions were eligible to be included in models based on the BMA approach. The BMA approach identified potential models for the study outcomes ‘appropriate medication transaction’, ‘antibiotic dispensing’, and ‘corticosteroid dispensing’ with the posterior probability values of 33.2%, 37.7%, and 36.6%, respectively (Table S2, available in Additional file 1). However, the probability values of all explanatory variables in the models for the outcomes ‘appropriate medication transaction’ and ‘corticosteroid dispensing’ were less than 50%. In the model for the outcome ‘antibiotic dispensing’, only the probability value of the explanatory variable ‘type of pharmacy’ was greater than 50%; therefore, the logistic regression was developed to explore the association between type of pharmacy and antibiotic dispensing (Table 4). CPs at chain pharmacies were less likely to dispense antibiotics compared to those at independent pharmacies, with OR of 0.29 (95% CI, 0.12–0.69).

**Table 4:** Factor associated with dispensing antibiotics

Type of pharmacy	Dispensing antibiotics				
	Yes (*N* = 56)	No (*N* = 38)	OR^a^	95% Cl	P value^b^
Chain	16 (28.6)	22 (57.9)	0.29	0.12–0.69	0.005
Independent	40 (71.4)	16 (42.1)			

^a^OR: adjusted odds ratio

^b^Logistic regression model was used to examine the relation between the variables and the dispensing of antibiotics

## Discussion

Our study found only 13.3% of medication transactions provided by CPs for a simulated COVID-19 patient with mild symptoms were deemed appropriate. Antibiotics and corticosteroids accounted for 59.2% and 60.2% of the transactions, respectively. Although the government has issued guidelines on COVID-19 treatment for home-based COVID-19 treatment and self-monitoring [5], these findings raise concern about medication misuse in COVID-19 treatment among community pharmacies in Vietnam.

Over half (59.2%) of the recorded transactions dispensed by CPs comprised at least one antibiotic. It should be noted that dispensing antibiotics without a prescription in community pharmacies is prohibited under the laws of Vietnam [11, 28]. However, this practice in Vietnam has been reported in previous studies before and after the COVID-19 pandemic [24, 29–31]. Additionally, an increase in antibiotic dispensing by community pharmacies has been documented in other regions during the pandemic [32]. Possible explanations for this practice include misconceptions about the effectiveness of antibiotics in treating COVID-19, pressure to fulfill patient expectations and insufficient regulatory control and enforcement of prescription-only policies [11, 33, 34]. Antibiotic overuse in COVID-19 treatment may result in adverse effects as well as antimicrobial resistance [35, 36]. For this reason, targeted interventions - such as enhanced regulatory inspection, continuous professional training, and public awareness campaigns - are needed to avoid long-term consequences, given that recent national data indicate a high prevalence of multidrug-resistant organisms in Vietnam [37].

Another issue of concern was the irrational dispensing of corticosteroids for COVID-19 patients with mild symptoms. According to current guidelines on COVID-19 treatment, corticosteroids can be used only if the patients develop severe or critical symptoms [38–40]. In addition, a recent study has shown that using corticosteroids at an early stage in mild to moderate COVID-19 patients can potentially worsen patient’s clinical conditions [41]. Nonetheless, 60.2% of the surveyed CPs dispensed corticosteroids for the simulated COVID-19 patient in our study without any further instructions. Overuse of corticosteroids was also reported among CPs in Vietnam before the pandemic due to misbelief, commercial pressures and weak regulatory enforcement [42]. This practice raises concern regarding potential risks for COVID-19 patients with mild symptoms and may reflect gaps in adherence to COVID-19 treatment guidance among CPs.

Our findings also provided insight into how CPs in HCMC responded to the COVID-19 pandemic. Most CPs were aware of the typical symptoms of COVID-19; however, 54.9% of them provided general advice on COVID-19 to the SCs. Furthermore, less than 10% of the surveyed CPs provided guidance on COVID-19 emergency warning signs concerning the appropriate timing of taking corticosteroids. Noticeably, around 7% of the CPs provided information on medications dispensed, all of which focused on possible adverse effects of molnupiravir. It should be mentioned that all pharmacies included in the studies voluntarily participated in the COVID-19 healthcare campaign in HCMC [13]. Their participation suggests that these pharmacists were actively involved in providing COVID-19-related services and support, potentially receiving some level of training or updates on COVID-19 management. The observed disparities between information provided and medications dispensed by the CPs may warrant interventions to improve knowledge, skills and attitude towards the healthcare needs for COVID-19 patients [43].

One notable finding of this study was the association between the type of a pharmacy and the antibiotic dispensing practice in CPs. It was noted that the rate of antibiotics dispensed in chain pharmacies was lower than that in independent ones. This finding is consistent with previous research conducted in India (2017) and Pakistan (2022) which indicated that independent pharmacies were more likely to dispense medications without a prescription compared with chain ones [44, 45]. This may be because most chain pharmacies focus on establishing strong and trustworthy brand image by improving customer experience as well as working with experts to enhance capabilities of their pharmacists whereas independent pharmacies tend to prioritize medicine sales.

### Strengths and limitations

While other studies examining pharmaceutical care provision during the COVID-19 pandemic through interviews and surveys [46, 47], this study used the SC method, a robust alternative to prevent biases related to actual behaviors of the pharmacists. In addition, each pharmacy was visited by two SCs to avoid recall bias. Although this might affect the consistency of implementing the scenarios, the use of standardized script could mitigate this issue.

However, some information about community pharmacists might be challenging to gather using the SC method. For instance, less than 10% of surveyed pharmacists disclosed their educational attainments as many were unwilling to share personal details; therefore, we decided not to report this information in our study. In addition, the study was limited to pharmacies in 13 central districts of HCMC that participated in the COVID-19 healthcare campaign, which may not be representative of all pharmacies in Vietnam. The identification of unlabelled medications relied solely on the assistance of a CP with more than 10 years of experience; however, some medications could not be accurately identified due to the absence of packaging or labeling information. However, unlabelled medications may compromise patient safety by hindering proper use and continuity of care, emphasizing the need for strengthened regulatory monitoring. Moreover, the constraints related to COVID-19 pandemic, time and research costs limited the scale of this study. For this reason, further and follow-up studies in HCMC and other provinces across Vietnam are recommended.

## Conclusions

This study highlighted a notably low proportion of transactions deemed appropriate for a patient with mild COVID-19 symptoms, along with inadequate counseling information provided by CPs. Despite these challenges, it is important to acknowledge the vital role that pharmacists play in healthcare, particularly in Vietnam, where pharmacies are often the most accessible and the first point of contact for patients seeking medical advice and treatment. Our findings underscores the urgent need to review and enhance pharmacist training programs and strengthen regulations on medication dispensing, particularly antibiotics and corticosteroids during the pandemic. Such efforts will not only improve the management of COVID-19 but also contribute to a safer, more effective, and sustainable healthcare system in the future.

## Supplementary Information

Below is the link to the electronic supplementary material.

Supplementary Material 1

## Data Availability

No datasets were generated or analysed during the current study.

## Abbreviations

CPs: Community pharmacists
HCMC: Ho Chi Minh City
SC: Simulated client
WHO: World Health Organization
IQR: Interquartile Range
