# Supplementary material for: Practices of community pharmacists towards COVID-19 treatment in Vietnam: a simulated client study
Source: BMC Health Serv Res. 2026 Aug 18;26:1602. doi: 10.1186/s12913-026-15395-0 (PMC13760818; doi:10.1186/s12913-026-15395-0)
Supplement: Supplementary file 1 [file 12913_2026_15395_MOESM1_ESM.docx]

## **Additional file 1:**

**Table S1. Medications dispensed by community pharmacists**

| **Medications dispensed** | | **n (%)** |
| --- | --- | --- |
| Antipyretics  (N=88) | Paracetamol | 8 (9.1) |
|  | Paracetamol + caffeine/epinephrine | 9 (10.2) |
|  | Paracetamol + cough medicines/antihistamines H1 | 25 (28.4) |
|  | Paracetamol effervescent tablet | 4 (4.6) |
|  | Paracetamol + ibuprofen | 1 (1.1) |
|  | Paracetamol + dextromethorphan + phenylephrine + guaiphenesin + vitamin C | 1 (1.1) |
|  | Recommended* | 26 (29.6) |
|  | Unlabelled** | 14 (15.9) |
| Dietary supplements  (N=78) | Multi-vitamin | 22 (28.2) |
|  | Zinc | 3 (3.8) |
|  | Multi-vitamin + Zinc | 13 (16.7) |
|  | Thymomodulin | 1 (1.3) |
|  | Recommended* | 21 (26.9) |
|  | Unlabelled** | 18 (23.1) |
| Cough medicines  (N=92) | Essential oils for cough | 26 (28.3) |
|  | Dextromethorphan HBr | 4 (4.4) |
|  | Codein | 1 (1.1) |
|  | Essential oils + dry cough medicines | 19 (20.7) |
|  | Dry cough medicines + wet cough medicines | 25 (27.2) |
|  | Cough suppressant (-dry cough) | 2 (2.2) |
|  | Essential oils + dry cough medicines  + wet cough medicines | 10 (10.9) |
|  | Terpin hydrate | 3 (3.3) |
|  | Unlabelled** | 2 (2.2) |
| Electrolyte supplements  (N=7) | Oresol | 2 (28.6) |
|  | Oresol-related supplement | 3 (42.9) |
|  | Recommended* | 2 (28.6) |
| Corticosteroids  (N=59) | Methylprednisolone | 26 (44.1) |
|  | Prednisolone | 3 (5.1) |
|  | Unlabelled** | 30 (50.9) |
| Antibiotics  (N=58) | Azithromycin | 10 (17.2) |
|  | Ciprofloxacin | 1 (1.7) |
|  | Levofloxacin | 1 (1.7) |
|  | Cefixime | 6 (10.4) |
|  | Cefuroxime | 4 (6.9) |
|  | Cefadroxil | 2 (3.5) |
|  | Cefpodoxime | 1 (1.7) |
|  | Cefdinir | 1 (1.7) |
|  | Amoxicillin-clavulanic acid | 21 (36.2) |
|  | Amoxicillin-clavulanic acid + clarithromycin | 3 (5.2) |
|  | Ampicillin-sulbactam | 1 (1.7) |
|  | Recommended* | 1 (1.7) |
|  | Unlabelled** | 6 (10.4) |
| Antiviral drugs  (N=7) | Molnupiravir | 7 (100) |
| Antacids  (N=7) | Omeprazole | 7 (100) |
| Antihistamines | Fexofenadine/cetirizine/levocetirizine | 7 (100) |

*****Medications that the pharmacists recommended but the SCs refused to buy to save on cost.

****** Unlabelled medications were visually identified by an experienced pharmacist based on appearance and packaging.

**Table S2. Selected model based on BMA approach**

| **Explanatory variables** | **Probability associated with 100% appropriate medicine dispensed** | **Probability associated with dispensing corticosteroids** | | **Probability associated with dispensing antibiotics** | |
| --- | --- | --- | --- | --- | --- |
| Type of the pharmacy | 31.6% | | 31.5% | 93.7% |  |
| Sex | 30.6% | | 19.7% | 7.2% |  |
| Identification | 16.5% | | 26.9% | 39.9% |  |
| Age | 7.4% | | 5.7% | 10.0% |  |
| Consultation length | 7.8% | | 4.3% | 10.6% |  |
| Intercept | -1.83 | | 0.39 | 2.15 |  |
| Number of variables | 0 | | 0 | 1 |  |
| BIC | -346.98 | | -295.68 | -299.25 |  |
| Probability model | 33.2% | | 36.6% | 37.7 |  |
| Regression coefficient | | | | -1.23 |  |
